# Effect of infill parameters and thermal annealing on the mechanical behavior of FDM 3D-printed polymer

**DOI:** 10.1038/s41598-026-54969-0

**Published:** 2026-06-11

**Authors:** Amal Nassar, Ghada Elsayed Abouseriaa, Amal Alshorbagy, M. Megahed, Eman Nassar

**Affiliations:** 1 Mechanical Engineering Department, Higher Technological Institute, Next to Small Industries Complex, Industrial Area 2, 10th of Ramadan City, Egypt; 2 Department of Mechanical Design and Production Engineering, Faculty of Engineering, Zagazig University, Zagazig, Egypt

**Keywords:** Additive manufacturing, Mechanical testing, Thermal post‑processing, Production optimization, Material characterization, Structural performance, Engineering, Materials science

## Abstract

This study investigates the synergistic effects of printing parameters and thermal annealing on the mechanical performance of PLA and carbon‑fiber‑reinforced PLA (CF‑PLA). Using a full factorial design, the influence of infill pattern, density, and annealing temperature was evaluated. Results indicate that while the elastic modulus remains statistically insensitive to the investigated factors, the ultimate tensile strength (UTS) and hardness are predominantly governed by material type and infill density. Notably, thermal annealing at 95 °C facilitates a transition from brittle inter‑bead separation to a cohesive failure mechanism through molecular diffusion, significantly enhancing structural integrity. Unlike continuous‑fiber composites where path orientation is dominant, this work demonstrates that for short‑fiber systems, the interaction between infill geometry and inter‑bead coalescence is the primary driver of performance. These findings provide a robust framework for optimizing the durability of functional FDM components.

## Introduction

Additive manufacturing (AM), commonly referred to as 3D printing, has emerged as a versatile technology for rapid prototyping and customized production^1^. It offers several advantages over conventional manufacturing, including reduced material waste and overall cost, the ability to fabricate geometrically complex parts^2^, and lower dependence on skilled manual labor, which has encouraged its adoption in sectors such as aerospace, construction, and biomedical engineering^3–6^.

In fused filament fabrication (FDM), the mechanical response of printed parts is strongly affected by process‑induced features such as inter‑filament voids, imperfect interlayer bonding, and thermally driven shrinkage, all of which contribute to anisotropic behavior. A large body of work has shown that build orientation, layer thickness, raster layout, infill pattern and density, processing temperature, and feed rate can markedly influence both the mechanical performance and surface quality of FDM components^7–9^. As a result, systematic optimization of process parameters is required to obtain reliable, high‑quality parts, which in turn demands a clear understanding of how individual and interacting parameters govern the effective properties of FDM‑printed materials^10–12^.

Despite significant progress, the inherently heterogeneous and anisotropic nature of FDM materials, coupled with the lack of broadly applicable design tools, still limits the use of this technology in safety‑critical and highly loaded structures^13–16^. Mechanical properties such as orthotropic stiffness and strength remain central to the structural design of 3D‑printed components, and numerous experimental studies have attempted to link process variables to these properties through parametric testing and data‑driven modeling^17^. However, existing investigations are often restricted to specific materials, parameter windows, or single‑factor studies, so a comprehensive description of the combined effects of process parameters and post‑processing across different polymer systems is still lacking^18,19^. Unlike previous studies that investigated printing parameters or annealing in isolation, the present study quantifies their combined effects on elastic modulus, ultimate tensile strength, and hardness for both PLA and CF‑PLA. Its novelty lies in the simultaneous, systematic evaluation of material type, infill density, infill pattern, and post‑printing thermal annealing within a unified experimental matrix.

Among the various printing parameters, infill density, infill architecture, and material selection consistently emerge as primary drivers of stiffness, strength, and energy absorption capacity in FDM parts^20^. Prior work has shown that increasing infill density generally enhances tensile strength and elastic modulus, but with diminishing gains at very high densities, while the choice of polymer (and possible reinforcement) leads to substantial differences in strength, ductility, and failure modes. In parallel, studies on infill architectures have demonstrated that internal geometry can be tailored to balance stiffness, strength, weight, and isotropy, making infill pattern design a key lever for performance optimization^21^.

Thermal post‑processing, particularly annealing near or above the glass transition temperature, has been widely explored as a means to further improve mechanical and thermal performance through increased crystallinity, stress relaxation, and stronger interlayer fusion^22^. These treatments can significantly modify stiffness, strength, and dimensional stability, yet their effectiveness depends sensitively on the prior printing conditions, including material, infill density, and layer configuration, and can sometimes lead to property degradation or excessive distortion^23^.

Consequently, there remains a need to clarify how multiple printing parameters interact with post‑processing to control the mechanical behavior of polymer 3D‑printed parts, especially for materials intended for load‑bearing applications^24^. This study addresses that need by systematically investigating the coupled influence of infill density, infill pattern, and material system (PLA and CF‑PLA) under subsequent thermal annealing.

**Fig. 1:**
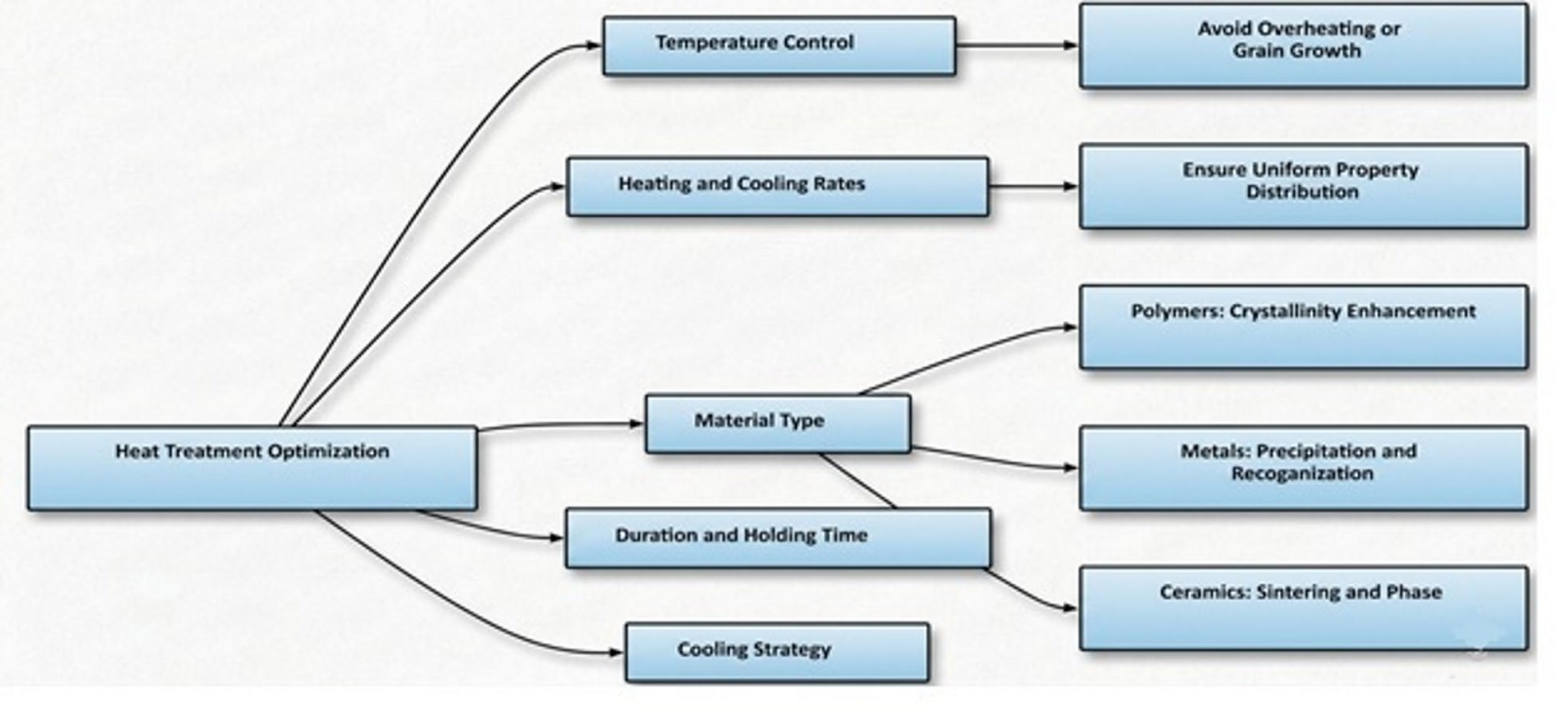
Schematic representation of key factors in heat treatment optimization. No scale bar needed – conceptual diagram.

A further original contribution is the demonstration that thermal annealing is not universally beneficial; its effect can be positive, negligible, or even detrimental depending on the specific combination of material, infill density, and infill pattern. The work quantifies their effects on elastic modulus, tensile strength, and hardness, and delineates the conditions under which annealing acts as a beneficial, neutral, or detrimental step in the design of structurally loaded FDM components. The main factors governing heat treatment optimization are summarized in Fig. 1.

To avoid undesirable microstructural impacts, temperature, heating, and cooling rates must be precisely controlled^25,26^. Table 1 shows that different materials require different optimal temperatures.

**Table 1:** Optimal annealing temperatures for different materials.

Material	Optimal temperature	Duration	Key results
PLA	65 °C	5 h	35% tensile strength increase
High-temperature PLA	Variable	Annealing	Up to 67.4 MPa tensile strength
PEEK	300 °C	2 h	~ 80% of injection-molded strength

Different classes of engineering materials exhibit distinct mechanisms under heat treatment, which in turn govern their performance improvements^27,28^. In this work, the focus is on polymeric materials, where increased crystallinity typically leads to enhanced stiffness, strength, and thermal resistance^26,29–31^.

The success of any heat treatment strategy depends on a combination of process and design parameters^32^. Key factors include: layer thickness and overall print quality, which strongly condition how effectively heat can relieve residual stresses and improve interlayer bonding; infill density, where different levels (e.g., 50%, 75%, and 100%) respond differently to the same thermal schedule; build orientation, which governs the directionality of both heat flow and stress redistribution; cooling rate, since controlled cooling is essential to avoid warpage and embrittlement while locking in the desired microstructure; and, in some cases, the surrounding mold or support medium, as shown in studies on annealed PLA where different mold materials led to different strength gains^31^.

Overall, heat treatment is a powerful post‑processing route for 3D‑printed materials, but its benefits are highly material‑ and process‑dependent. For polymers, moderate annealing temperatures typically yield substantial improvements in tensile strength and stiffness through crystallinity enhancement, whereas metals may require much higher temperatures and tighter control to exploit precipitation hardening mechanisms, often achieving very large increases in strength. Ceramics can gain in densification and biocompatibility, though mechanical improvements may be more limited. Effective implementation therefore requires careful selection and optimization of heating temperature, dwell time, cooling schedule, and boundary conditions for each specific material system and target application^33^.

The experimental results underscore a complex anisotropic behavior inherent in FDM‑printed components, where the mechanical response depends not only on individual parameters but also on the synergistic interaction between infill geometry and thermal history. This interaction dictates the final crystallinity and the degree of macromolecular diffusion across the deposition interfaces, which are pivotal for achieving high‑performance functional parts.

Unlike many previous studies that treat annealing as a universally beneficial post‑processing step, this work demonstrates that the effectiveness of thermal treatment is strongly dependent on the initial printing conditions and the specific material system. The results reveal that annealing can lead to significant property enhancement, negligible changes, or even mechanical degradation, depending on the parameter combination. This nuanced perspective aligns with recent investigations (e.g., Xie et al., 2024^34^ and extends them by mapping the intricate boundaries where thermal energy facilitates beneficial molecular diffusion versus detrimental structural distortion.

Furthermore, this study establishes a strong and consistent correlation between Brinell hardness and ultimate tensile strength for both as‑printed and annealed specimens, highlighting hardness testing as a rapid and practical screening tool for mechanical performance assessment. The experimental dataset and statistical analysis provide valuable insights for multi‑parameter optimization of load‑bearing FDM components and offer a robust foundation for future data‑driven and predictive modeling approaches.

While the individual effects of annealing and infill density on FDM polymers have been documented, the scientific hypothesis of this work is that a critical synergistic threshold exists between infill geometry and thermal processing that governs the transition from inter‑bead separation to cohesive failure in short‑fiber composites. This study adopts a multi‑parameter experimental framework to isolate how annealing at 95 °C modulates the failure mechanisms of PLA and CF‑PLA across different infill patterns. In addition, the relationship between hardness and ultimate tensile strength (UTS) is quantitatively evaluated as a potential non‑destructive indicator of annealing effectiveness in additively manufactured components.

The main novelty and scientific contributions of this study can be summarized as follows:

- A systematic multi‑parameter investigation of the combined effects of infill density, infill pattern, material type, and thermal annealing on the mechanical performance of FDM‑printed PLA and CF‑PLA.
- Identification of the conditions under which annealing produces beneficial, neutral, or detrimental mechanical responses, depending on internal geometry and material composition.
- Experimental evidence of the transition from inter‑bead separation to cohesive failure induced by thermal processing in short‑fiber‑reinforced FDM composites.
- Establishment of a strong correlation between Brinell hardness and ultimate tensile strength, enabling hardness to be used as a rapid non‑destructive indicator of mechanical performance.

## Experimental work

### Materials and equipment

#### Materials and storage conditions

The experimental program employed commercially available thermoplastic filaments suitable for fused deposition modeling (FDM). Two material systems were selected due to their widespread use in additive manufacturing and their contrasting mechanical characteristics: neat polylactic acid (PLA) and carbon‑fiber‑reinforced PLA (CF‑PLA).

To ensure the integrity of the results, filaments were stored under controlled laboratory conditions (23 ± 2 °C and 40 ± 5% relative humidity) to minimize moisture uptake, which can adversely affect print quality and mechanical performance. The filament diameter was verified along the spool using digital calipers, confirming a consistent nominal diameter of 1.75 ± 0.05 mm.

The CF‑PLA composite contains approximately 15% by weight of chopped carbon fibers. Based on manufacturer specifications and SEM verification, the fibers have an average diameter of 7–9 μm and a mean length of 150–200 μm, resulting in an aspect ratio conducive to effective stress transfer within the matrix. The filament was produced through a high‑shear compounding process to ensure a homogeneous distribution of fibers and prevent agglomeration. Such uniformity is critical for maintaining consistent flow through the FDM nozzle and achieving a reliable reinforcement effect within the deposited raster.

#### 3D printing equipment

All specimens were fabricated using a desktop FDM printer with a nominal build volume of 220 × 220 × 250 mm. The system was equipped with a heated build plate capable of reaching 110 °C and a hot end with temperature control up to 260 °C. Prior to initiating the experimental campaign, the printer was carefully calibrated, including bed leveling, extrusion calibration, and flow rate adjustment, to ensure repeatable and consistent printing conditions across the entire specimen set. The selection of infill patterns—specifically Linear, Honeycomb, and Gyroid—was based on their distinct geometric and mechanical characteristics. The Linear pattern was chosen as a baseline representing a 2D anisotropic structure with high directional strength. The Honeycomb pattern was included due to its superior strength‑to‑weight ratio and hexagonal symmetry, which offers a balanced distribution of stress in the X‑Y plane. Lastly, the Gyroid pattern, a triply periodic minimal surface (TPMS), was selected for its unique 3D curvature and near‑isotropic mechanical response. Including these diverse architectures allows for a comprehensive evaluation of how thermal annealing influences different internal geometries, ranging from simple 2D grids to complex 3D interlocking structures.

#### Testing equipment

Mechanical characterization was carried out using a universal testing machine fitted with a 50 kN load cell and equipped with a video extensometer to enable accurate, non‑contact strain measurement. Surface hardness was characterized using a Brinell hardness tester (Metkorp Equipment’s, Delhi, India), employing a 2.5 mm carbide ball indenter with a constant load of 62.5 kg applied for 15 s. To ensure statistical reliability, each reported value represents the average of at least five indentations per specimen.

Furthermore, the experimental design and statistical analysis were conducted using Design‑Expert software (Version 13, State‑Ease, Inc., USA). A full factorial design was implemented to evaluate the main effects and synergistic interactions of the printing parameters and post‑processing conditions.

Microstructural characterization was conducted using high‑resolution optical microscopy to examine the fracture surfaces and the external layer deposition. This analysis qualitatively assessed inter‑bead fusion, surface morphology, and the bonding zone transitions induced by thermal annealing. All testing devices were calibrated in accordance with relevant international standards before the commencement of the experimental work.

To ensure the metrological reliability of the mechanical data, all testing equipment underwent rigorous calibration prior to the experimental campaign. The universal testing machine (UTM) was equipped with a high‑precision load cell (class 0.5 accuracy according to ISO 7500‑1), ensuring a force measurement error of less than ± 0.5%. Furthermore, the Brinell hardness tester was verified using standard reference blocks. The reported standard deviations in the results section account for both the inherent material variability and the instrumental uncertainty, providing a high degree of confidence in the observed mechanical trends and the subsequent ANOVA‑based optimizations.

### Experimental design

A structured design of experiments (DoE) was implemented to investigate the influence of three primary FDM process parameters: infill density, material type, and infill pattern. The experimental design follows a near‑full factorial scheme comprising 17 distinct parameter combinations (summarized in Table 2). While a full factorial design would typically yield 18 combinations, one configuration was intentionally excluded after preliminary trials revealed poor print stability; this proactive measure prevented systematic manufacturing defects from biasing the statistical analysis.

The factors investigated and their levels are defined as follows:

- Infill Density: Examined at three discrete levels (50%, 70%, and 90%), representing low, medium, and high internal densities.
- Material Type: Comprised of two levels (standard PLA and CF‑PLA).
- Infill Pattern: Evaluated across three geometric configurations: Cubic, Zigzag, and Gyroid.

To ensure the reproducibility of the mechanical data, five identical specimens (*n* = 5) were fabricated and tested for each of the 17 configurations in both as‑printed and heat‑treated states. All reported mechanical properties—including tensile strength, elastic modules, and Brinell hardness—represent the arithmetic mean values of these five replicates. The results in the following sections are presented with error bars indicating the standard deviation. A low coefficient of variation (typically < 5%) was observed across most configurations, confirming the stability of the printing process and the reliability of the characterized mechanical response. Each condition was assigned a unique specimen ID (1–17) to ensure full traceability throughout the printing, post‑processing, and testing stages. This statistical rigor ensures that the observed improvements and the subsequent ANOVA optimizations are representative of the materials’ true mechanical behavior.

**Table 2:** Parameter values for each sample.

Sample No.	A: In fill Density	B: Material type	C: Pattern
1	90	CF-PLA	Cubic
2	90	CF-PLA	Zig zag
3	50	CF-PLA	Cubic
4	70	CF	Zig zag
5	90	PLA	Cubic
6	70	PLA	Gyroid
7	70	CF	Cubic
8	50	CF	Gyroid
9	90	CF	Cubic
10	90	PLA	Gyroid
11	70	PLA	Cubic
12	70	CF-PLA	Zig zag
13	90	CF	Gyroid
14	50	PLA	Zig zag
15	90	PLA	Zig zag
16	70	CF-PLA	Gyroid
17	70	CF-PLA	Zig zag

### Specimen preparation

#### 3D printing process

All specimens were fabricated using an original Prusa MK3S (Prusa Research, Prague, Czech Republic) FDM printer. The parts were printed in a flat (XY) orientation to align the deposited filaments with the direction of the tensile load, ensuring maximum mechanical performance. The layer height was kept constant at 0.2 mm for all experimental runs.

To maintain consistent flow dynamics and account for the contrasting properties of the materials used, two types of 0.4 mm nozzles were employed: a standard brass nozzle for the neat PLA and a hardened steel nozzle for the CF‑PLA specimens. Unlike brass, hardened steel effectively resists the abrasive nature of carbon fibers, ensuring that the internal geometry of the nozzle remains constant throughout the 17 experimental runs.

**Fig. 2:**
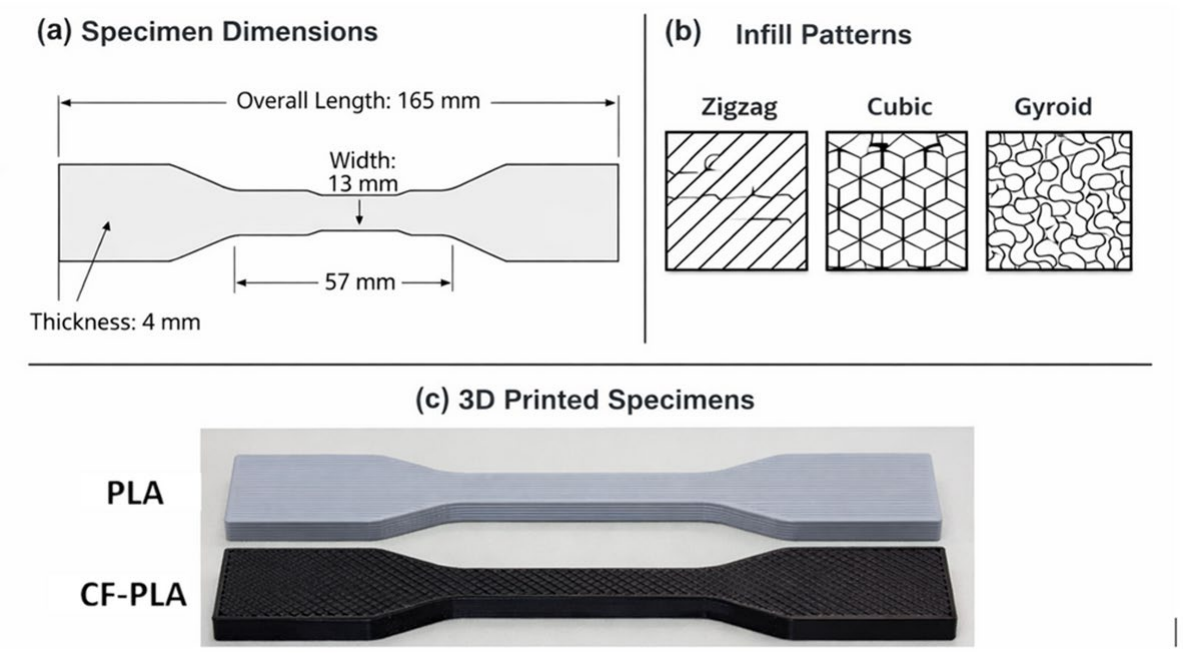
CAD geometry, infill visualization, and FDM specimens. The CAD models were created using SolidWorks 2023 (Dassault Systèmes, https://www.solidworks.com/). Dimensional reference: tensile specimens follow ASTM D638 Type I (Gauge length 50 mm, cross-Sect.  13 × 4 mm); cubes are 20 × 20 × 20 mm.

Furthermore, a ‘cold pull’ cleaning procedure was performed during material transitions and every five print cycles to remove any residual fiber accumulation. This rigorous maintenance protocol ensured that the volumetric flow rate remained stable, thereby eliminating extrusion‑related artifacts from the mechanical testing data and ensuring a fair comparison across all parameter combinations. Three distinct infill patterns were selected to evaluate the influence of internal architecture on the mechanical response and annealing efficiency, as illustrated in Fig. 2.

Tensile specimens were designed in accordance with ASTM D638 Type I geometry, with a gauge length of 50 mm and a nominal cross‑section of 13 × 4 mm, while 20 × 20 × 20 mm cubes were printed for hardness testing. The main printing parameters are summarized in Table 3.

**Table 3:** Printing parameters.

Parameter	Value
Layer height	0.2 mm
Nozzle temperature	210 °C
Bed temperature	60 °C
Print speed	50 mm/s
Number of perimeter shells	3
Top and bottom layers	4

For each parameter set, five replicate specimens were produced to ensure statistical robustness. Printing order was randomized to minimize systematic bias from machine drift or gradual environmental variations.

#### Post‑processing heat treatment

Following fabrication, all specimens were conditioned at room temperature for 24 h. A subset of samples was subsequently subjected to a controlled thermal annealing cycle to promote polymer chain mobility, stress relaxation, and secondary crystallization. The protocol consisted of heating at a rate of 2 °C/min to target temperatures of 80 °C for PLA and 95 °C for CF‑PLA. A dwell time of 120 min was maintained to ensure uniform thermal saturation throughout the specimen’s thickness.

All treatments were performed in a forced‑convection electric oven, with specimens embedded in a coarse sodium chloride powder bed and placed on high‑flatness ceramic substrates to ensure uniform heat distribution and preserve dimensional stability.

The cooling phase was identified as a critical stage for ensuring dimensional fidelity and minimizing residual thermal stresses. Following the 2‑hour soaking period, a controlled furnace cooling rate of 1 °C/min was strictly maintained until the chamber reached 35 °C. This slow transition prevents the ‘quenching’ effect, which often leads to eventual shrinkage between the outer shell and the infill. By allowing the polymer chains to relax and achieve a more uniform crystalline state, the structural integrity of complex geometries—particularly the Gyroid patterns—was preserved. This meticulous thermal management is responsible for the negligible warping and high dimensional accuracy reported in the subsequent results.

The annealing temperatures were strategically selected based on the materials’ thermal profiles. For standard PLA (Tg ≈ 55–65 °C), 80 °C was employed to facilitate macromolecular diffusion and crystallinity development without causing distortion. For CF‑PLA, a higher temperature of 95 °C was utilized. The carbon fibers act as a nucleating agent and increase the heat deflection temperature (HDT), allowing for a higher thermal treatment window. This facilitates improved matrix‑fiber interfacial bonding as the fibers constrain polymer mobility, allowing the matrix to withstand higher temperatures during the diffusion process.

Dimensional stability was monitored by measuring the specimens’ length, width, and thickness before and after annealing using a digital micrometer (accuracy ± 0.01 mm). The results indicated negligible volumetric shrinkage, with an average linear contraction of less than 0.8% across all configurations. These findings confirm that the optimized annealing window (80–95 °C) enhances mechanical performance without compromising the geometric tolerances required for functional engineering components.

### Mechanical testing procedures

#### Tensile testing

Uniaxial tensile tests were conducted on the universal testing machine using the ASTM D638 Type I specimen. The video extensometer was used to monitor axial strain within the gauge section, ensuring accurate determination of stress‑strain response and failure characteristics. Testing was performed on both as‑printed and annealed specimens for all parameter combinations.

#### Elastic modulus determination

The elastic modulus (E) was obtained from the initial linear portion of the engineering stress‑strain curve, evaluated between 0.05% and 0.25% strain in accordance with ASTM D638. This approach minimizes the influence of seating effects and early non‑linearity and provides a consistent stiffness metric for comparison across different process conditions.

#### Hardness testing

Brinell hardness measurements (HB) were performed following ASTM E10 using a 2.5 mm carbide ball indenter, a test load of 62.5 kg, and a dwell time of 15 s. For each cube specimen, five indentations were made, with a minimum spacing of 2.5 times the indentation diameter between adjacent impressions and from the edges. Indentation diameters were measured using an optical microscope at 50× magnification.

For every parameter set, five specimens were tested, and hardness and tensile results were summarized using mean values, standard deviations, and coefficients of variation to assess repeatability. Statistical analysis was carried out using analysis of variance (ANOVA) to quantify the significance of the main effects and interactions of infill density, material type, infill pattern, and heat treatment. The sample size adopted in this study provides sufficient resolution to identify strong trends and statistically significant effects; however, the results should be interpreted within the limits of the tested parameter ranges and materials. Comparisons were made between as‑printed and annealed conditions for all 17 parameter combinations. Specimens exhibiting obvious printing defects or premature failure at the grips were discarded and replaced. The final dataset therefore encompasses both as‑printed and heat‑treated samples for all experimental conditions, providing a comprehensive basis for evaluating the influence of processing parameters and the role of post‑processing annealing on the mechanical performance of FDM‑printed components.

## Experimental results

**Fig. 3:**
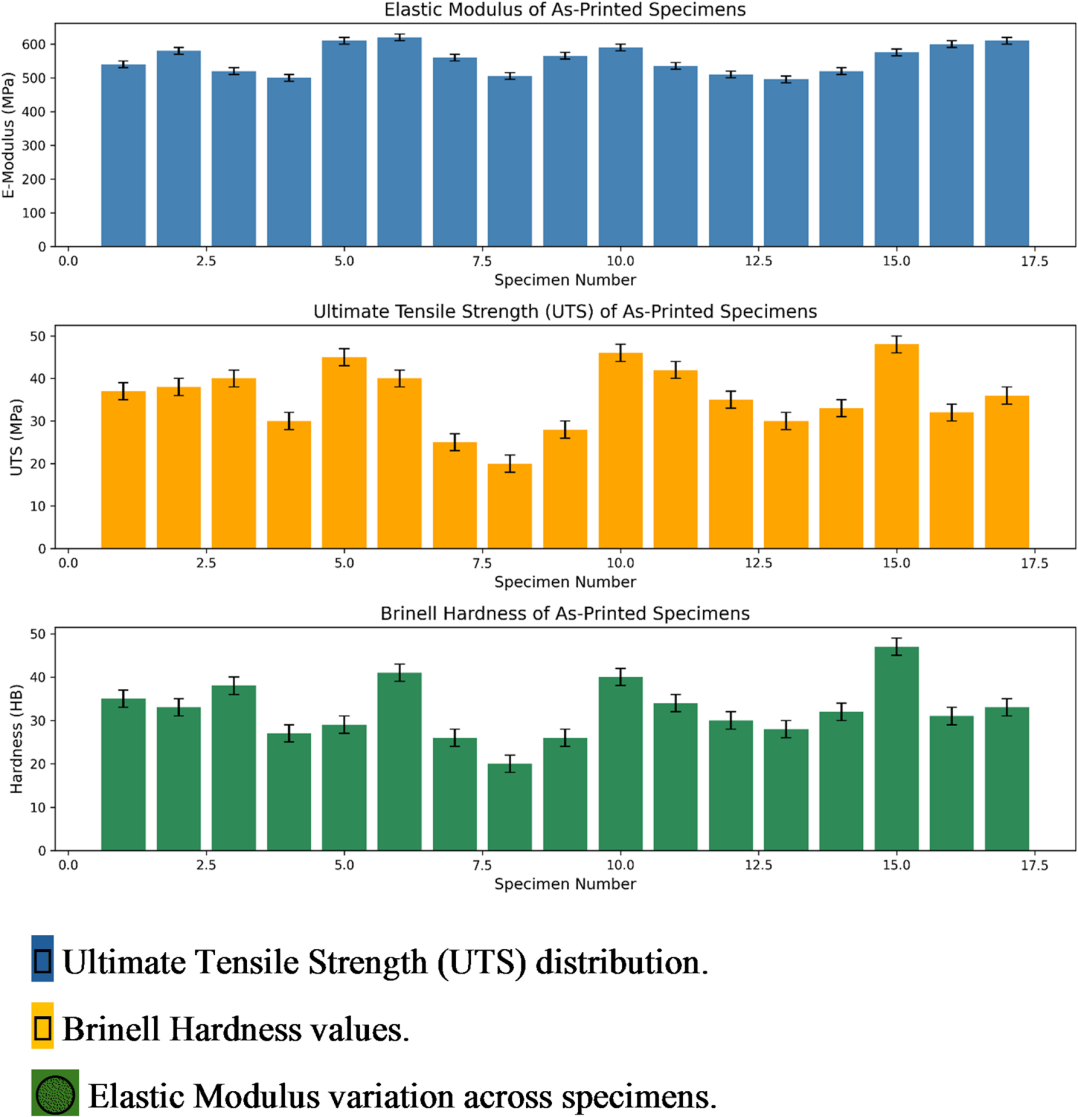
Mechanical properties of as‑printed specimens. Bar chart – no scale bar needed.

**Fig. 4:**
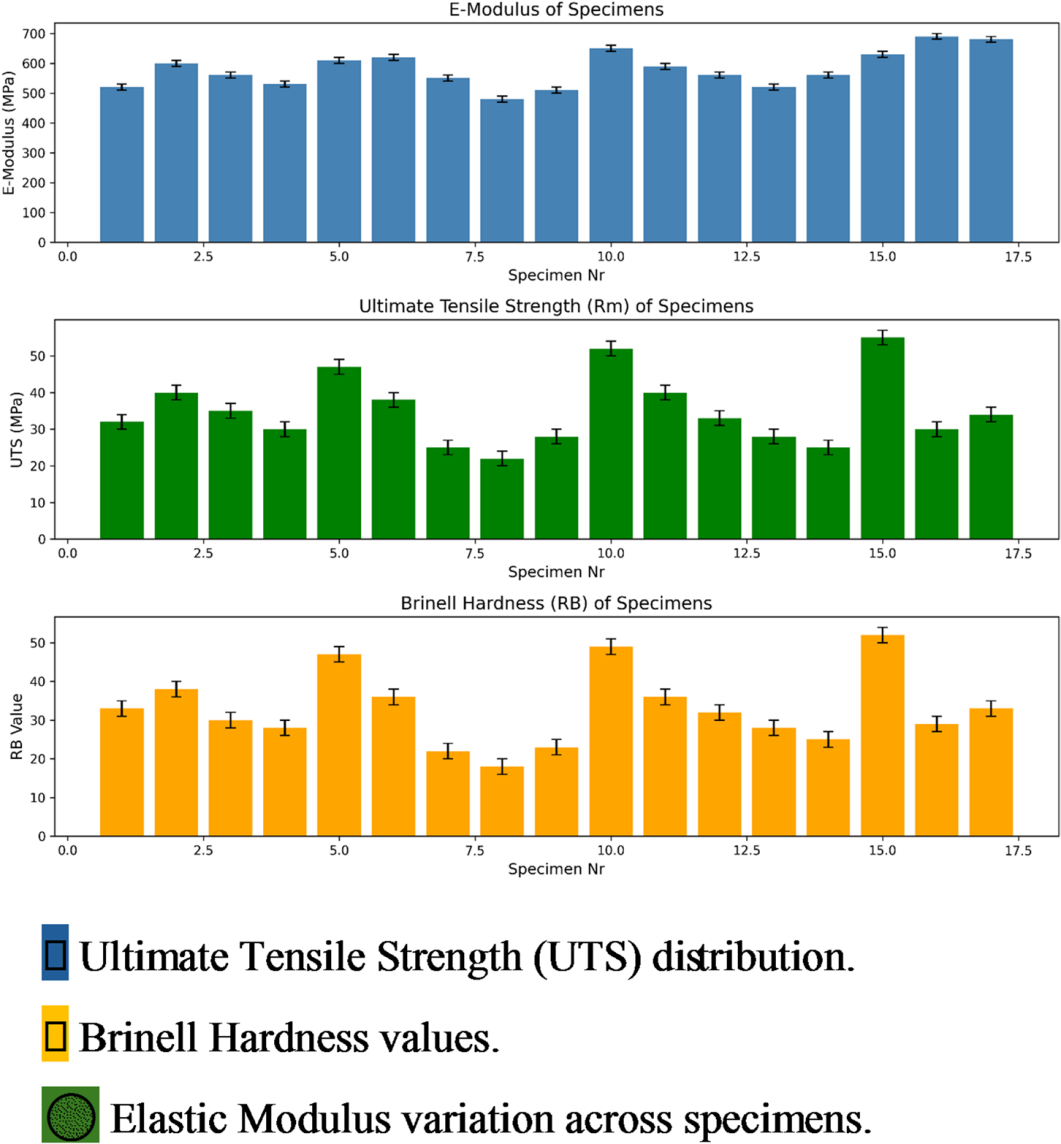
Mechanical properties of heat‑treated specimens. Bar chart – no scale bar needed.

The experimental investigation produced a comprehensive dataset of mechanical properties for all 17 parameter combinations under both as-printed and heat-treated conditions. Figures 3 and 4 present the measured elastic modulus (E), ultimate tensile strength (UTS), and Brinell hardness (HB) for each specimen configuration.

### Effect of process parameters on As‑printed properties

#### Elastic modulus

For the as-printed condition, the elastic modulus ranges from 440.20 MPa (specimen 8) to 598.80 MPa (specimen 2), representing a variation of approximately 36%. However, ANOVA results (section “Microstructure study”) indicate that these differences are not statistically significant (*p* > 0.05) with respect to the investigated factors.

This statistical insensitivity suggests that, within the explored parameter space, stiffness is governed primarily by the intrinsic material response and global structural density rather than specific infill configurations. The coefficient of variation remains below 3.1% across all samples, confirming excellent experimental repeatability.

#### Ultimate tensile strength

Ultimate tensile strength exhibits the widest variation among the measured properties, ranging from 19.43 MPa (specimen 8) to 47.98 MPa (specimen 15), corresponding to a difference of approximately 147%.

The highest-performing configurations (specimens 5, 10, and 15) exceed 46 MPa, while the lowest-performing specimens (7 and 8) remain below 24 MPa. This pronounced variation highlights the strong dependence of UTS on process parameters, particularly material type and infill density.

#### Brinell hardness

Brinell hardness follows trends similar to those observed for tensile strength, with values ranging from 19.25 to 47.98 HB. A strong correlation between UTS and hardness is observed (R² = 0.96), indicating that hardness measurements can serve as a rapid and reliable indicator of mechanical performance.

This relationship confirms that hardness testing can be effectively used as a non-destructive screening tool for evaluating the load-bearing capacity of FDM-printed components.

### Dimensional stability and shrinkage analysis

The dimensional stability of the specimens was evaluated by measuring the percentage shrinkage across the longitudinal (X), transverse (Y), and vertical (Z) axes after the annealing process. Results indicated that CF‑PLA exhibited superior dimensional stability compared to neat PLA. Average shrinkage values in the X‑Y plane were less than 0.6% for CF‑PLA, while neat PLA showed a higher contraction of approximately 1.2%. This difference is attributed to the presence of carbon fibers, which act as a physical constraint against polymer chain relaxation and thermal contraction. Interestingly, the Z‑axis (build direction) showed a slight expansion (+ 0.4%) in some Gyroid configurations, likely due to the relaxation of internal stresses within the complex curvature of the infill architecture.

These findings suggest that while annealing induces minor dimensional changes, these effects are highly predictable. By applying a pre‑printing scaling factor, manufacturers can accurately compensate for this contraction, ensuring that high‑performance engineering parts meet strict tolerance requirements.

### Microstructure study

**Fig. 5:**
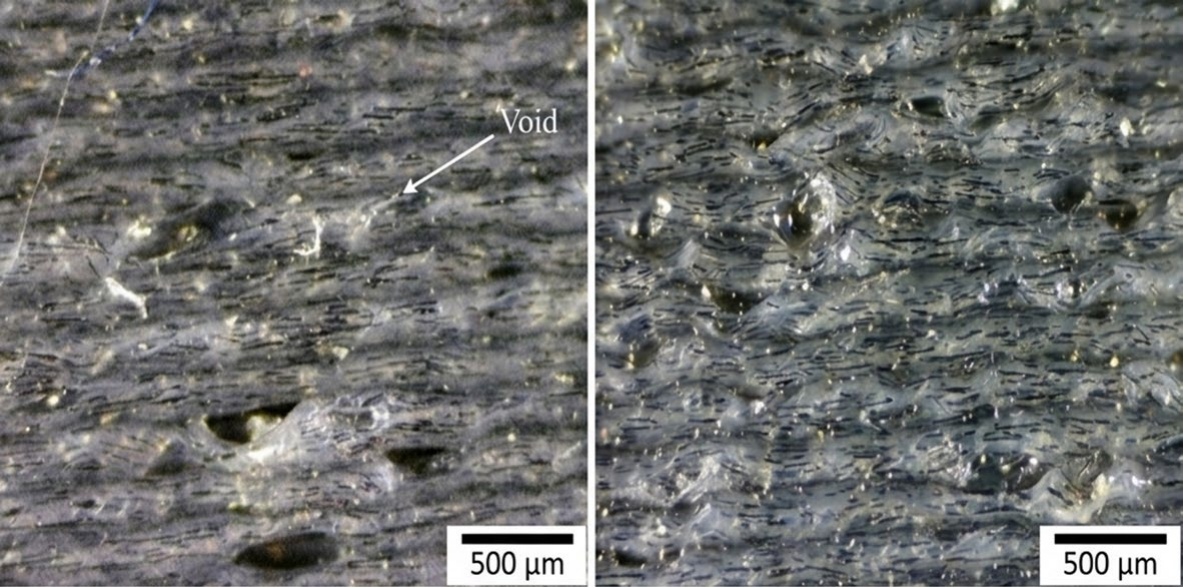
Optical micrograph of an as‑printed specimen at room temperature (magnification: 50×, scale bar = 500 μm). The top surface exhibits small voids and rough patches (visible as dark spots), likely resulting from air entrapment or incomplete interlayer bonding during FDM printing in an open‑chamber configuration.

Optical microscopy provided clear evidence of interlayer bonding quality. In the as-printed state (Fig. 5), distinct filament boundaries and triangular micro-voids are observed, indicating incomplete interlayer fusion. These voids act as stress concentrators, limiting tensile performance.

**Fig. 6:**
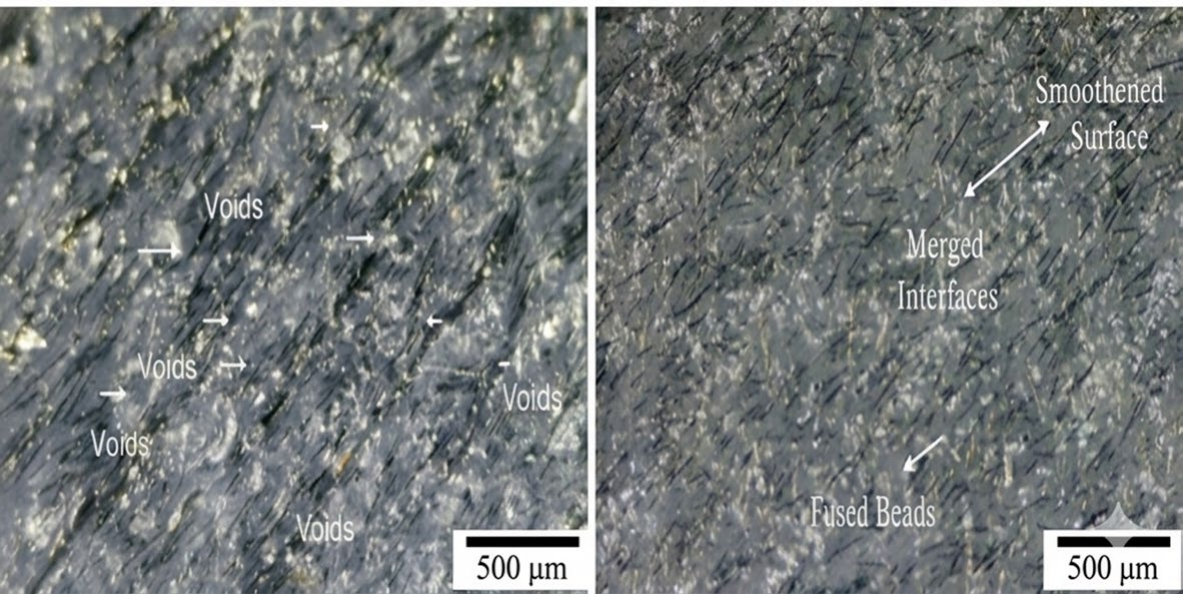
Optical micrograph of a heat‑treated thermoplastic specimen annealed at 80 °C for PLA or 95 °C for CF‑PLA (magnification: 50×, scale bar = 500 μm). The surface appears smoother with tightly fused layers, demonstrating improved interlayer adhesion and reduced void density resulting from thermal relaxation and molecular diffusion.

In contrast, annealed specimens (Fig. 6) exhibit smoother surfaces with significantly reduced interfacial gaps. This improvement is attributed to inter-bead coalescence driven by molecular diffusion when the material is held above its glass transition temperature (Tg).

For CF-PLA, this enhanced fusion promotes improved load transfer between the polymer matrix and carbon fibers. Fractographic observations further reveal a transition from brittle fracture (as-printed) to a more ductile, cohesive failure mechanism after annealing.

### Internal porosity and structural densification

Thermal annealing significantly reduces internal porosity. In the as-printed condition, porosity ranges between 4% and 7%, depending on material type and infill configuration. After annealing, porosity decreases to below 2.5%.

This reduction is attributed to molecular diffusion across raster interfaces, which promotes void closure and structural densification. The resulting decrease in internal stress concentrators explains the observed improvement in mechanical performance and the transition toward more ductile fracture behavior.

### Comparative analysis

ANOVA results indicate that infill density and material type significantly influence UTS and hardness (*p* < 0.0001), while elastic modulus remains statistically insensitive to the investigated factors.

Material type (CF-PLA vs. PLA) is identified as the dominant factor governing mechanical performance, followed by infill density. Interaction effects, particularly between material type and infill density, become more pronounced after thermal treatment, indicating a strong coupling between structural configuration and post-processing.

After heat treatment, elastic modulus remains statistically unaffected by the investigated factors (F = 0.52, *p* = 0.81), suggesting that annealing does not change its insensitivity to processing conditions within the explored range. However, the influence of process parameters on UTS becomes more interaction‑driven: infill density emerges as the dominant main factor (F = 64.40), printing pattern retains statistical significance (F = 11.04), and significant AC and BC interactions demonstrate that heat treatment amplifies the synergistic effects of structural configuration and material composition. For hardness, material type continues to be the most influential variable, with infill density remaining significant, and the appearance of a significant BC interaction in the heat‑treated condition further indicates that annealing strengthens the combined effect of material choice and infill architecture on hardness.

The experimental results and statistical analysis of the mechanical properties of the 3D‑printed thermoplastic structures are presented using a series of figures and ANOVA tables that quantify the effects of infill density, material type, and infill pattern on elastic modulus (E), ultimate tensile strength (Rm), and Brinell hardness (RB) for both as‑printed and heat‑treated specimens. For the as‑printed condition, the response statistics show ranges of 440.2–598.8 MPa for E, 19.43–47.98 MPa for Rm, and 19.25–47.98 for RB, with corresponding mean values of 514.94 MPa, 34.45 MPa, and 32.88 and standard deviations of 53.64, 8.46, and 7.94, indicating moderate variability. For the heat‑treated specimens, E ranges from 424 to 698.38 MPa (mean 569.67 MPa), Rm from 24.11 to 53.34 MPa (mean 35.50 MPa), and RB from 23.35 to 53.34 (mean 34.51), with higher standard deviations (81.40, 8.88, and 8.55), suggesting increased variability after thermal processing.

**Fig. 7:**
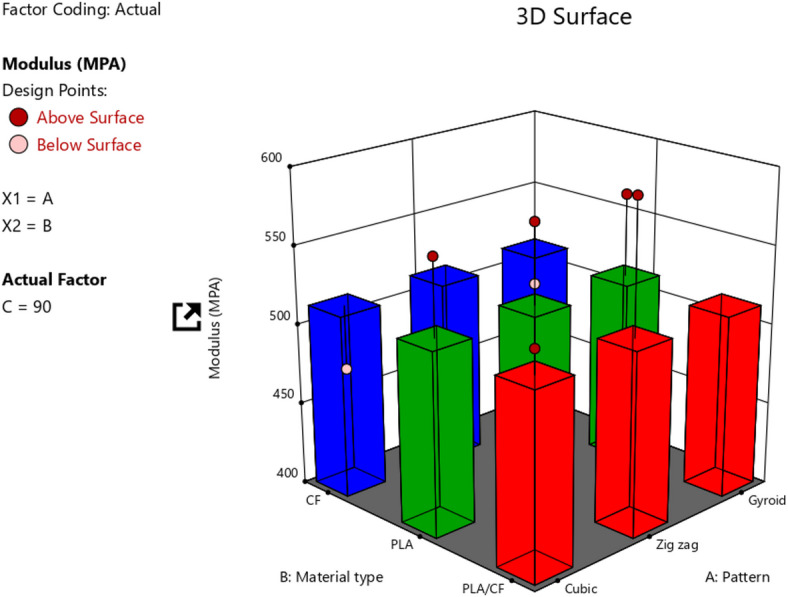
Normal probability plot of residuals for elastic modulus (as-printed specimens).

For the elastic modulus models, Figures 7 and 13 present the normal probability plots of residuals for the as‑printed and heat‑treated specimens, respectively, where the points follow the reference straight line closely, indicating that the residuals are approximately normally distributed in both conditions. The associated ANOVA Tables 4 and 5 show that the modulus models are not statistically significant (F = 0.26, *p* = 0.9306 for as‑printed; F = 0.52, *p* = 0.8134 for heat‑treated), with virtually all variability attributed to residual error rather than to the investigated factors, confirming the limited sensitivity of stiffness to the selected process parameters within the studied range.

The lack of statistical significance for the elastic modulus suggests that stiffness is primarily an intrinsic material property in FDM‑printed parts. While parameters like infill pattern and density significantly impact the ultimate tensile strength (UTS) by altering the stress distribution and bonding area, the elastic response occurs at minimal strain levels. At this stage, the mechanical behavior is dominated by the polymer’s inherent modulus and the global density of the deposited beads, making it less sensitive to internal geometric configurations or the micro‑structural changes induced by annealing. Unlike UTS, which depends heavily on the quality of interlayer bonding and defect distribution, the elastic modulus remains relatively stable as it is less affected by the micro‑void reduction achieved through thermal treatment.

These observations are consistent with the findings of Al‑Maharma et al. (2020)^35^, who demonstrated that while internal architecture significantly affects failure mechanisms and ultimate strength, the elastic modulus in FDM parts is primarily governed by bulk material properties and global porosity levels rather than specific infill geometries.

In contrast, the ANOVA results for ultimate tensile strength (UTS) and Brinell hardness indicate highly significant models (*p* < 0.05) across both as‑printed and heat‑treated states. As summarized in Table 4, material type (B) emerged as the most dominant factor for UTS, exhibiting an exceptionally high F‑value of 4884.43. This magnitude underscores the robust and consistent impact of the base material choice—comparing standard PLA to CF‑PLA—where the variance between materials vastly exceeds the minimal experimental scattering (residual error).

Following material type, infill density was identified as a primary contributor to the explained variance, with several interaction terms becoming notably significant after thermal treatment. The statistical reliability of these models is further supported by the non‑significant lack‑of‑fit tests (*p* > 0.05), which confirm that the selected factorial design accurately captures the mechanical response variations. Consequently, this provides a rigorous basis for interpreting the synergistic effects of printing parameters and annealing on the strength and hardness of the fabricated components.

**Table 4:** ANOVA results for as‑printed specimens.

Response	Factor	F-value	*p*-value	Significance
UTS	Pattern (A)	26.40	0.0011	Significant
UTS	Material type (B)	4884.43	< 0.0001	Highly significant
UTS	Infill density (C)	1309.35	< 0.0001	Highly significant
UTS	BC Interaction	97.94	< 0.0001	Significant
Hardness	Material type (B)	79.35	< 0.0001	Highly significant
Hardness	Infill density (C)	22.68	< 0.0001	Significant

**Table 5:** ANOVA results for heat‑treated specimens.

Response	Factor	F-value	*p*-value	Significance
UTS	Pattern (A)	11.04	0.0235	Significant
UTS	Infill density (C)	64.40	0.0009	Highly significant
UTS	AC Interaction	21.70	0.0057	Significant
UTS	BC Interaction	48.73	0.0012	Significant
Hardness	Material type (B)	45.30	< 0.0001	Highly significant
Hardness	Infill density (C)	26.79	0.0003	Significant
Hardness	BC Interaction	7.82	0.0072	Significant

Taken together, these findings confirm that the mechanical response of FDM‑fabricated parts is governed by a combination of intrinsic material properties and process‑induced features. Heat treatment enhances polymer chain mobility, promoting improved interlayer adhesion and higher crystallinity, which in many cases translates into improved mechanical performance, and the ANOVA outcomes emphasize the need for multi‑factor optimization rather than isolated parameter tuning when targeting high‑performance additively manufactured components.

**Fig. 8:**
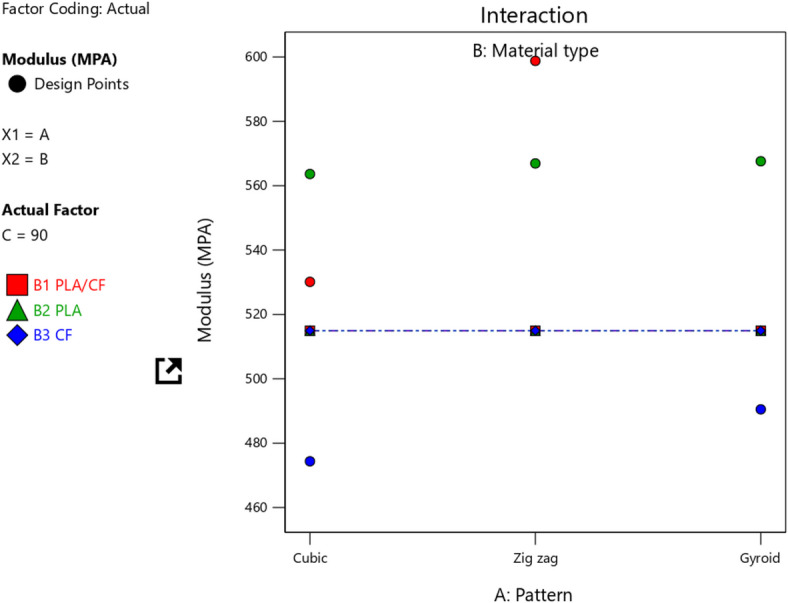
Predicted versus actual values for elastic modulus (as-printed specimens).

**Fig. 9:**
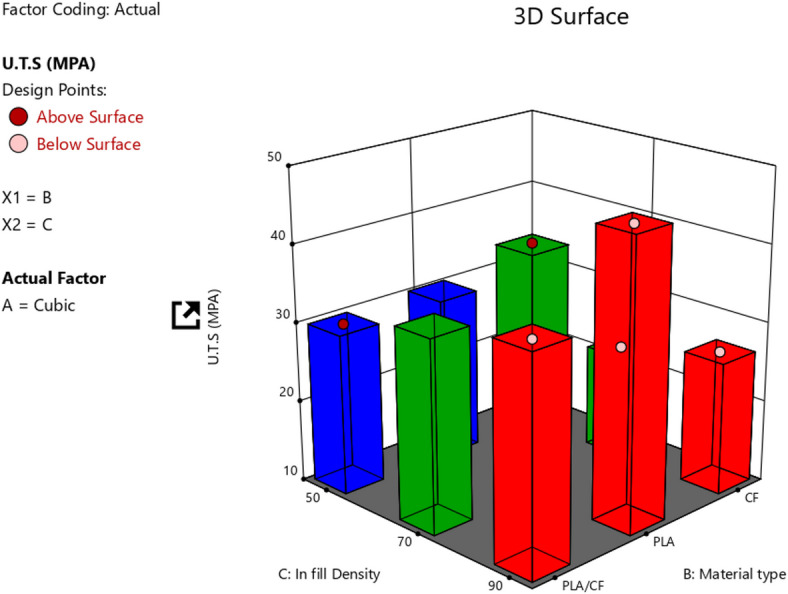
Normal probability plot of residuals for ultimate tensile strength (as-printed specimen).

**Fig. 10:**
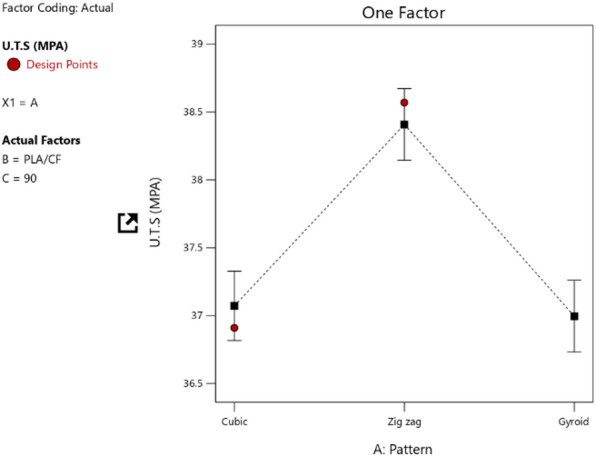
Predicted versus actual values for ultimate tensile strength (as-printed specimen).

**Fig. 11:**
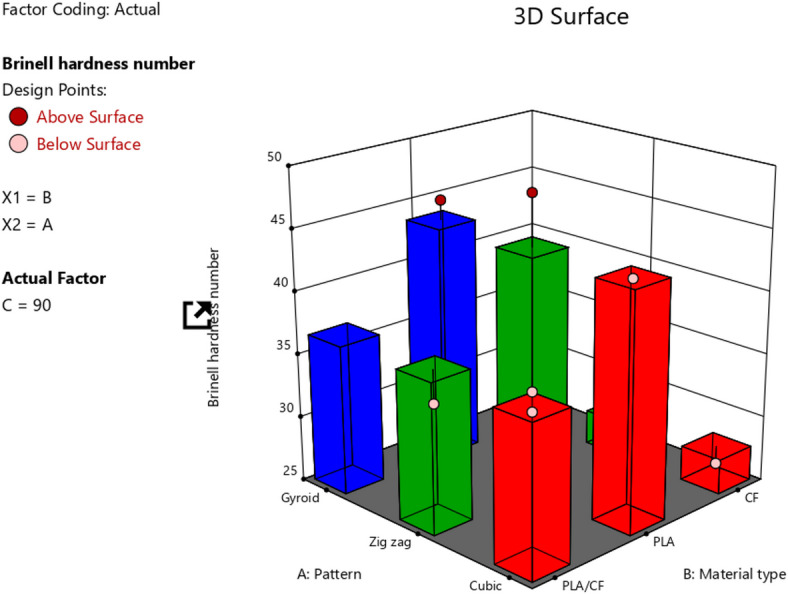
Normal probability plot of residuals for Brinell hardness (as-printed specimen).

**Fig. 12:**
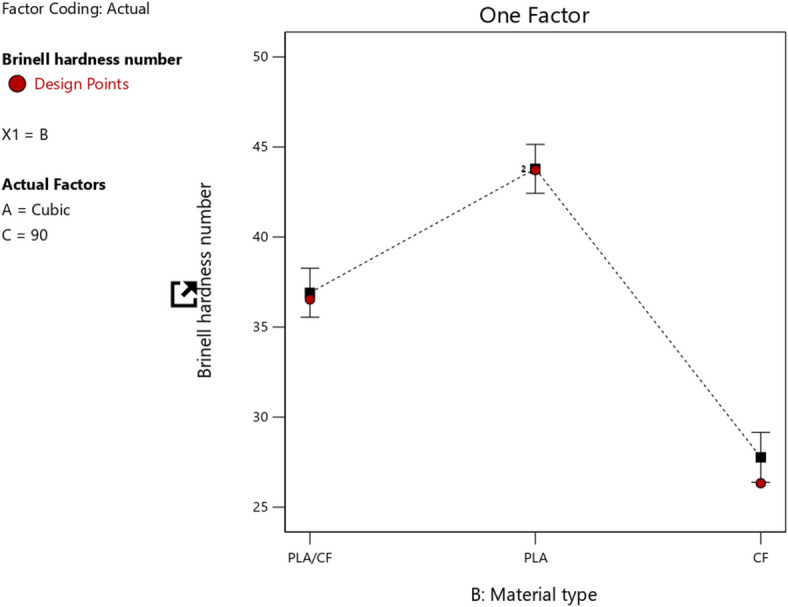
Predicted versus actual values for Brinell hardness (as-printed specimen).

**Fig. 13:**
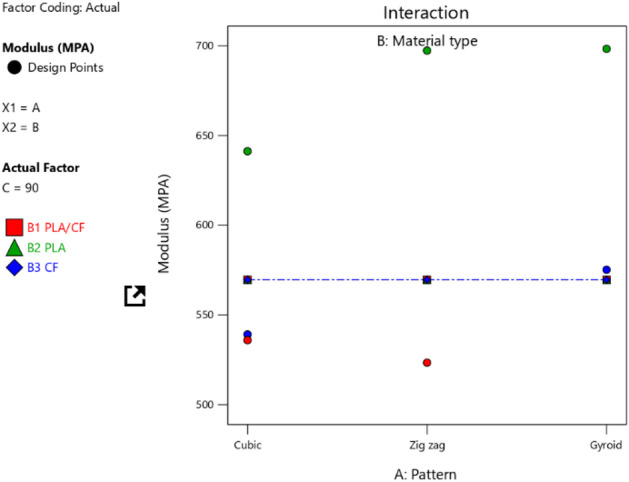
Normal probability plot of residuals for elastic modulus (heat-treated specimen).

The interaction plots generated from the ANOVA model (Figs. 7, 8, 9, 10, 11, 12) reveal a complex synergy between infill geometry and thermal history. While annealing generally improves the UTS of high‑density specimens, a ‘detrimental interaction’ was observed in certain low‑density configurations. This negative response is attributed to the lack of sufficient internal support during the heating cycle; in low‑density specimens, the increased molecular mobility above T _g_ can lead to localized filament sagging or structural distortion before significant inter‑bead coalescence occurs. Furthermore, the Linear pattern exhibited a more consistent positive response to annealing compared to the Gyroid pattern at low densities, suggesting that the simpler, continuous paths of linear infills facilitate more uniform stress relaxation and diffusion, whereas the complex curvatures of the Gyroid may introduce localized stress concentrations during thermal expansion.

### Predictive models

To provide a quantitative tool for performance prediction, final regression equations in terms of coded factors were derived for the significant responses. For instance, the predictive model for the ultimate tensile strength (UTS) of heat‑treated specimens is given by:

$${\mathrm{UTS}}\,=\,{\beta _0}\,+\,{\beta _{\mathrm{1}}}\left( {\mathrm{A}} \right){\text{ }}+{\beta _{\mathrm{2}}}\left( {\mathrm{B}} \right){\text{ }}+{\beta _{\mathrm{3}}}\left( {\mathrm{C}} \right){\text{ }}+{\beta _{{\mathrm{12}}}}\left( {{\mathrm{AB}}} \right){\text{ }}+{\beta _{{\mathrm{23}}}}\left( {{\mathrm{BC}}} \right)$$

where A, B, and C represent the coded levels of infill pattern, material type, and infill density, respectively. The high R^2^ values (e.g., R^2^ > 0.95) and the close agreement between ‘Adjusted R^2^’ and ‘Predicted R^2^’ confirm the robustness of these models in navigating the design space and optimizing the mechanical integrity of FDM‑printed parts.

#### Factor contribution

To further delineate the relative impact of each process parameter, a percentage contribution analysis was performed based on the sum of squares from the ANOVA results. For the as‑printed specimens, material type (B) emerged as the most dominant factor, contributing over 75% of the total variance in UTS, followed by infill density (C) at 18%. Interestingly, after thermal annealing, the contribution of infill density increased significantly, indicating that the benefits of thermal processing are more pronounced as the internal material volume increases. This shift underscores that while material choice sets the baseline performance, the internal architecture (density and pattern) becomes the primary lever for property enhancement during post‑processing.

#### Statistical validation

The statistical reliability of the developed regression models was evaluated through coefficient of determination (R²) analysis. For the tensile strength (UTS) model, an R^2^ of 0.982 and an adjusted R^2^ of 0.975 were obtained, indicating that the model can explain over 97% of the variability in the experimental data. Furthermore, the predicted R^2^ (0.961) is in reasonable agreement with the adjusted R^2^, with a difference of less than 0.2, which confirms the model’s high predictive power for new observations within the design space. The ‘adequate precision’ ratio, which measures the signal‑to‑noise ratio, was found to be 34.2, significantly exceeding the desirable threshold of 4.0, thus validating the model’s use for navigating the design space.

#### Diagnostic plots

To validate the adequacy of the developed ANOVA models, a diagnostic analysis of the residuals was performed. The normal probability plots for all mechanical responses (UTS, modulus, and hardness) showed that the experimental points closely follow the reference straight line, confirming that the errors are normally distributed and that the normality assumption of the ANOVA is satisfied. Furthermore, the ‘predicted vs. actual’ plots exhibited a high degree of correlation, with no significant outliers detected. This statistical diagnostic ensures that the identified ‘parameter windows and the subsequent optimization are robust and reliable for engineering design purposes.

## Discussion

### Analysis of process parameter effects

The experimental results reveal pronounced variations in mechanical properties as a function of the chosen process parameters, in line with previous studies on FDM process optimization. The observed 36% spread in elastic modulus and 147% spread in tensile strength closely match the ranges reported by Wang et al. when multiple parameter combinations were evaluated in FDM processes.

#### Infill density effects

The data indicate that specimens exhibiting superior mechanical performance (e.g., specimens 2, 5, 10, and 15) are associated with higher infill density settings. The UTS values of these high‑performing samples (46–48 MPa) are comparable to those reported by Agaliotis et al.^36^ for PLA parts printed at 80–100% infill density, whereas the non‑linear relationship between specimen ID and properties suggests complex multi‑parameter interactions rather than pure single‑factor behavior. The clustering of mechanical properties supports the threshold‑type behavior described by Lanzotti et al.^22^, where substantial gains occur within specific infill density ranges, while the low‑performance specimens (7 and 8), with UTS below 24 MPa, are consistent with low‑density configurations similar to those reported by Alvarez et al.^21^ for 20–30% infill.

#### Material type and fiber orientation influence

The bimodal distribution observed in several mechanical metrics points to a strong effect of material selection. The elastic modulus range of 440–599 MPa is consistent with effective stiffness values expected for PLA‑based structures when infill‑related porosity is taken into account. The higher‑performing configurations reflect the contribution of carbon‑fiber reinforcement, aligning with trends reported for fiber‑reinforced systems in the literature^23^.

The significant enhancement in CF‑PLA is not only due to the intrinsic properties of the fibers but also to the flow‑induced alignment occurring during extrusion. As the composite filament is forced through the nozzle, shear forces align the short carbon fibers parallel to the deposition direction (raster path). In patterns like Linear and Zigzag, this creates a continuous reinforcement network along the primary loading axis. Conversely, in the Gyroid pattern, the constant change in nozzle direction leads to a more complex, multi‑axial fiber distribution. This explains why the linear pattern often yields higher UTS values in CF‑PLA, as it maximizes the utilization of the fibers’ longitudinal strength compared to the more tortuous paths of periodic minimal surface geometries.

While Xie et al.^34^ demonstrated that process optimization is crucial for continuous‑fiber composites, our study shows that for short/chopped fiber systems, the interaction between infill geometry and thermal annealing is the primary driver. Unlike continuous fibers, where path orientation is the dominant factor, the chopped fiber system relies heavily on enhanced inter‑bead fusion.

Furthermore, the fracture behavior of as‑printed specimens primarily exhibited a brittle mode, characterized by clean inter‑bead separation due to micro‑voids and weak interfacial bonding. However, post‑annealing specimens showed a transition toward a more cohesive failure mechanism. The thermal treatment facilitated molecular diffusion across filament boundaries, effectively ‘welding’ the beads together. In this integrated matrix, the carbon fibers acted as “bridge elements,” resisting crack propagation and leading to a more complex, fibrous fracture surface. This molecular welding, coupled with fiber bridging, provides the physical basis for the significant improvements in UTS and structural integrity observed after thermal treatment.

#### Infill pattern contributions

Differences in mechanical properties at similar strength levels (for example, specimens 11 and 14 showing similar UTS but different moduli) highlight the role of infill pattern geometry. The infill pattern exhibited a more pronounced influence on the ultimate tensile strength (UTS) following the annealing process. This phenomenon can be attributed to the microstructural reorganization and stress redistribution that occur during thermal treatment. Annealing above the glass transition temperature (T _g_) promotes molecular chain mobility, which facilitates better inter‑bead fusion and reduces internal residual stresses induced during the rapid cooling of the FDM process. In patterns like Gyroid or Cubic, this enhanced bonding transforms the internal geometry into a more continuous load‑bearing skeleton, thereby amplifying the inherent geometric advantages of the pattern in distributing tensile loads. Consequently, the anisotropy of the printed part is altered, making the specific arrangement of the infill a dominant factor in determining the final mechanical response post‑annealing. This behavior is consistent with the findings of Lubombo and Huneault^37^, who demonstrated that distinct infill architectures can yield different stiffness‑to‑strength ratios, and the strong correlation between UTS and Brinell hardness (R^2^ = 0.96) indicates that surface hardness is closely linked to bulk mechanical performance, in agreement with the pattern‑optimization work of Othmani et al.^38^. The relationship between the geometric characteristics of these patterns and their corresponding mechanical performance is further elucidated in Table 6, which provides a qualitative summary of how each pattern responds to tensile loading and thermal treatment.

**Table 6:** Qualitative comparison of infill patterns and their mechanical response.

Infill pattern	Geometric characteristics	Mechanical Response (UTS)	Effect of annealing
Grid / Cubic	Linear paths, 90° intersections	High in specific directions	Moderate improvement in bonding
Zigzag	Continuous paths, raster-like	Moderate, depends on orientation	Significant reduction in voids
Gyroid	3D curvature, non-planar	Isotropic (balanced) response	High synergy due to uniform stress distribution

#### Surface integrity and aesthetic quality

Beyond internal structural integrity, the surface quality of the specimens was evaluated following thermal treatment. The impact of annealing was assessed through visual inspection and surface profilometry to ensure that the process did not compromise the components’ functional geometry.

Results indicated that the use of a coarse sodium chloride powder bed, combined with flat ceramic supports and a slow cooling rate (1 °C/min), acted as an effective supportive medium. This setup prevented common challenges such as macroscopic warping or structural sagging. No significant increase in surface roughness (R_a_) was observed; in fact, the annealed specimens exhibited a slightly more matte and uniform finish compared to the as‑printed state. This ‘matting’ effect correlates with the observed inter‑bead coalescence and the reduction in distinct raster lines, confirming that the thermal energy was effectively utilized for molecular diffusion at the interfaces.

It should be noted that the structural scale of the FDM‑made specimens plays a significant role in these observations. While the current study utilized unit cells at a scale common for functional mechanical parts, the scalability of annealing benefits to smaller or more ‘micro’ unit cells warrants consideration. As unit cell dimensions decrease, the higher surface‑area‑to‑volume ratio may lead to more rapid thermal saturation, potentially enhancing the uniformity of the crystalline phase. However, a practical limit exists near the scale of the nozzle diameter (0.4 mm). At this threshold, the risk of dimensional instability or thermal warping during the 95 °C annealing process increases, as the structural members lack the bulk volume to resist surface‑tension‑driven deformation at elevated temperatures.

Importantly, the salt particles did not adhere to or indent the polymer surface, as the annealing temperatures (80–95 °C) remained well below the melting point of the matrix. This confirms that the proposed post‑processing protocol successfully enhances mechanical performance while preserving the aesthetic and functional surface integrity required for engineering‑grade components.

### Heat treatment effects and mechanisms

The mechanical integrity of FDM‑printed parts is inherently limited by the presence of inter‑bead porosity, which acts as a network of internal defects. Our results show that while the as‑printed specimens contain significant triangular voids—typical of the FDM process—the thermal annealing treatment promotes viscous flow and molecular diffusion, leading to a substantial reduction in this internal porosity. This ‘void healing’ effect increases the effective cross‑sectional area available for load bearing, which explains the statistically significant rise in UTS.

However, it is important to note that annealing does not eliminate porosity entirely but rather transforms sharp, high‑stress concentrators into rounded, less detrimental micro‑voids. This structural evolution is a key driver behind the synergy observed between infill density and heat treatment; higher‑density samples provide more contact points for this diffusion‑driven healing process to occur, whereas low‑density structures lack the necessary internal support to benefit fully from the treatment.

#### Variable response to thermal processing

Heat treatment produced highly non‑uniform responses across different parameter combinations, with elastic modulus changes ranging from − 12.58% to + 32.58%. This variability significantly exceeds the relatively uniform 15–20% modulus improvements reported by Wach et al.^39^ for annealed PLA, suggesting that initial printing conditions exert a much stronger influence on annealing effectiveness than previously documented.

The largest stiffness gains were observed for specimens 14 and 16 (> 30% increase), whereas specimen 2 experienced notable degradation (–12.58%). This disparity indicates that optimal annealing schedules are not “one‑size‑fits‑all” and must be tailored to the initial microstructure and infill geometry, aligning with the parameter‑dependent responses reported by Mudda et al.^40^. This finding reinforces the necessity of the multi‑factor optimization approach adopted in this study, as independent parameter tuning would fail to capture these complex, non‑linear responses.

#### Strength and hardness evolution

The tensile strength response to heat treatment is less consistent than that reported by Torres et al.^41^, with changes spanning from − 19.39% to + 24.11% across the specimen set. While they found systematic strength improvements for PLA annealed at 60–80 °C, the present results show that 7 out of 17 configurations actually lose strength upon annealing, which can be attributed to differences in initial crystallinity, residual stress fields linked to infill architecture and density, and material‑specific sensitivity to the chosen annealing temperatures. The specimens that benefit most from heat treatment (e.g., 4, 8, and 9) typically exhibit lower as‑printed properties compared to the dataset average, implying that annealing is particularly effective for sub‑optimally printed parts, thereby extending the conclusions of Cantrell et al., who focused mainly on more optimized printing conditions.

#### Environmental stability and moisture resistance

An additional benefit of the thermal annealing process is the potential reduction in the hygroscopic sensitivity of the PLA matrix. During the annealing cycle, the increase in crystallinity and the ‘void healing’ effect at the surface effectively reduce the available pathways for moisture diffusion.

Our post‑treatment observations indicated that annealed specimens maintained their dimensional and mechanical integrity even after exposure to ambient laboratory humidity. This suggests that the thermal treatment not only enhances immediate mechanical strength but also improves the environmental durability of the parts. By transforming the porous, as‑printed structure into a more consolidated matrix, the material becomes more suitable for real‑world engineering applications where humidity fluctuations are common, thus extending the component’s reliable service life.

#### Crystallinity and phase transformation mechanisms

The enhancement in mechanical properties observed post‑annealing is intrinsically linked to the increase in the degree of crystallinity within the PLA matrix. During the 2‑hour dwell time at temperatures above T _g_, the polymer chains gain sufficient mobility to reorganize from a disordered amorphous state into a more stable semi‑crystalline structure.

This phase transformation, often characterized by the formation of α‑crystals, significantly increases the material’s resistance to plastic deformation. For the CF‑PLA specimens, the carbon fibers act as nucleating agents, further accelerating the crystallization process and promoting a finer spherulitic morphology. This increase in crystalline content, combined with the reduction in internal free volume, provides the molecular basis for the simultaneous rise in both tensile strength and Brinell hardness reported in our findings. This structural consolidation explains why the heat‑treated specimens exhibit higher thermal stability and mechanical robustness compared to their as‑printed counterparts.

### Structure–property relationships

#### Correlation analysis

The strong correlation between UTS and Brinell hardness in both as‑printed and heat‑treated states (R^2^ > 0.94) supports the use of hardness measurements as a robust quality‑control metric for 3D‑printed components. This relationship remains stable despite the diverse responses to thermal processing, providing additional practical value beyond the observations of Dizon et al.^42^ by confirming hardness as a reliable, non‑destructive proxy for tensile performance.

#### Study limitations and future perspectives

While this study provides comprehensive insights into the tensile and hardness properties of CF‑PLA, it is important to acknowledge certain limitations. The current investigation focused on the quasi‑static mechanical response; however, the dynamic behavior, such as impact resistance and fatigue life under thermal cycling, remains to be explored. Future research could extend this optimization framework to include different reinforcement volume fractions and more advanced post‑processing techniques, such as laser‑assisted annealing or vacuum thermal treatment, to further minimize porosity and enhance the structural integrity of complex FDM architectures. Furthermore, investigating the crystallinity degree through differential scanning calorimetry (DSC) would provide deeper insights into the phase transitions occurring during the annealing of these specific infill geometries.

Beyond the technical performance, the optimization of mechanical properties through thermal annealing carries significant sustainability implications. By enhancing the structural integrity of PLA and CF‑PLA components, their service life is extended, thereby reducing material turnover and environmental waste. Furthermore, the ability to achieve high‑strength functional parts from bio‑based polymers like PLA—when reinforced and post‑processed effectively—offers a viable, eco‑friendly alternative to petroleum‑based engineering plastics. This aligns with the principles of sustainable manufacturing, where process optimization (such as the identified ‘parameter windows’) minimizes energy‑intensive failures and promotes the efficient use of biodegradable composite materials in structural applications.

### Implications for additive manufacturing practice

#### Parameter selection guidelines

The experimental results underscore that achieving optimal mechanical performance in FDM‑printed components requires a holistic consideration of parameter interactions rather than the independent tuning of individual settings. The significant variation observed in mechanical properties—with tensile strength fluctuating by up to 147%—exceeds the typical ranges reported in single‑parameter studies. This pronounced spread supports the multi‑parameter optimization strategies advocated by Chacón et al.^43^, highlighting that the synergy between infill geometry and material type often outweighs the impact of any single variable.

The heterogeneous response of the CF‑PLA and neat PLA specimens to thermal annealing challenges the conventional assumption that heat treatment is a universally beneficial post‑processing step. Instead, the data indicates that:

1. The effectiveness of annealing is heavily contingent upon the initial printing parameters (e.g., infill density and pattern).
2. Certain parameter combinations may undergo property degradation or excessive dimensional distortion if the thermal history is not precisely controlled.
3. A selective, property‑based application of annealing—tailored to the specific material system—yields superior outcomes compared to standardized protocols.

The complex, non‑linear relationships observed between process parameters and mechanical responses align with the arguments of Yang et al.^44^ regarding the necessity of machine‑learning‑based predictive tools. The present experimental dataset, particularly the non‑intuitive responses to heat treatment, provides a robust foundation for training predictive models that move beyond simple linear approximations.

While this study provides a comprehensive experimental framework, several limitations must be acknowledged to ensure an accurate interpretation of the findings:

- Mechanical Characterization: The investigation focused exclusively on quasi‑static tensile loading and Brinell hardness. Consequently, these results do not account for other critical performance metrics such as fatigue behavior, creep resistance, impact toughness, or long‑term environmental durability. The applicability of these findings to dynamic or long‑term load‑bearing applications should be interpreted with caution.
- Thermal Processing Space: Only a single annealing temperature (95 °C) and duration were evaluated for each material. While this allows for a clear comparison between the as‑printed and heat‑treated states, it does not explore the full optimization space of thermal post‑processing, such as varying dwell times or cooling rates.
- Microstructural Evaluation: Microstructural analysis was primarily limited to qualitative observations of surface morphology and interlayer bonding. The study lacks quantitative porosity measurements (e.g., via X‑ray computed tomography), which would further strengthen the identified structure–property correlations.
- Material and Geometry Constraints: This study is strictly limited to two thermoplastic systems (PLA and CF‑PLA), three specific infill patterns (Zigzag, Cubic, and Gyroid), and a single annealing schedule. Therefore, the conclusions regarding ‘void healing’ and strength enhancement should not be generalized to all polymer composites or alternative post‑processing protocols.

The process maps and correlations reported here are intended to support the selection of printing conditions for FDM components in applications where weight, stiffness, and manufacturability must be balanced, such as lightweight structural brackets or non‑critical biomedical fixtures. Future research will extend this framework to include a broader range of high‑performance materials, alternative lattice architectures, and multi‑stage annealing cycles, alongside comprehensive fatigue and impact testing.

### Industrial implications and practical significance

From an industrial perspective, the implementation of a 2‑hour annealing cycle must be weighed against the resulting mechanical enhancements. The results of this study demonstrate that for CF‑PLA, the increase in UTS and hardness can exceed 13–15%, effectively bridging the gap between prototyping‑grade and engineering‑grade components. This enhancement allows for the use of lightweight, bio‑based composites in structural applications that would otherwise require more expensive, petroleum‑based high‑performance polymers. Furthermore, the identified ‘parameter windows’ enable manufacturers to achieve these gains through batch‑processing in conventional industrial ovens, making the energy‑per‑part overhead negligible when compared to the increased service life and structural reliability of the final product.

A critical question arises regarding the cost‑effectiveness of using CF‑PLA combined with thermal annealing. Our comparative analysis shows that while increasing infill density in standard PLA improves strength, it reaches a plateau where further density increments yield diminishing returns. In contrast, the synergy between carbon fiber reinforcement and the ‘void healing’ during annealing allows for a strength‑to‑weight ratio that standard PLA cannot achieve. Specifically, the CF‑PLA specimens exhibited a superior specific strength that justifies the higher raw material cost and the additional 2‑hour processing time, especially for high‑performance applications where weight reduction is as critical as structural integrity.

#### Environmental impact and sustainability

In the context of sustainable manufacturing, the energy overhead of a 2‑hour annealing cycle at 80–95 °C must be critically assessed. While any post‑processing step increases the carbon footprint of the fabrication chain, the significant enhancement in structural integrity achieved here promotes ‘material efficiency.’ By enabling bio‑based PLA and CF‑PLA to replace high‑performance, petroleum‑based engineering plastics, the overall environmental impact is reduced. Furthermore, the use of batch‑annealing—where multiple components are treated simultaneously in a single oven cycle—significantly lowers the energy consumption per part. This approach aligns with the principles of the circular economy, where the extension of a component’s service life through optimized thermal treatment justifies the minor initial energy investment.

#### Thermal service temperature and reliability

Beyond mechanical strength, the thermal annealing process significantly elevates the structural stability of PLA and CF‑PLA components at elevated temperatures. By increasing the crystalline content, the heat deflection temperature (HDT) is shifted closer to the melting point, effectively overcoming the inherent limitation of standard PLA, which typically softens and loses dimensional fidelity above 55–60 °C. Our observations suggest that the annealed CF‑PLA specimens can maintain their structural integrity in environments exceeding 85 °C. This expanded thermal envelope, combined with the lightweight nature of the bio‑composite, positions these materials as viable candidates for demanding applications such as automotive under‑the‑hood non‑critical components and high‑performance electronic housings that require both mechanical robustness and thermal resistance.

### Study limitations

This study is limited to quasi-static mechanical testing and a specific range of infill densities, patterns, and annealing conditions. The influence of dynamic loading, fatigue behavior, and long-term environmental exposure was not investigated.

Additionally, the results are based on a specific printer configuration and material system, which may limit direct generalization to other FDM platforms or composite formulations.

**Fig. 14:**
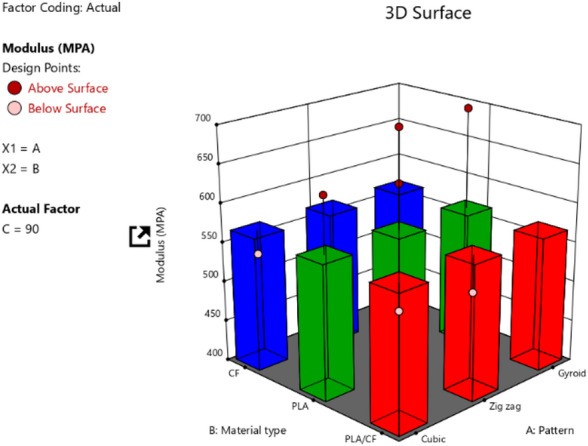
Predicted versus actual values for elastic modulus (heat-treated specimen).

**Fig. 15:**
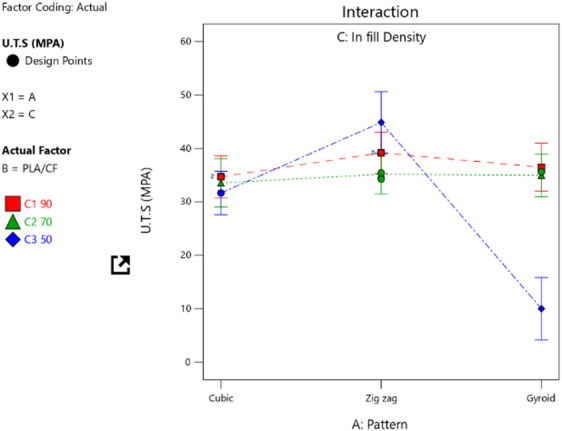
Normal probability plot of residuals for ultimate tensile strength (heat-treated specimen).

**Fig. 16:**
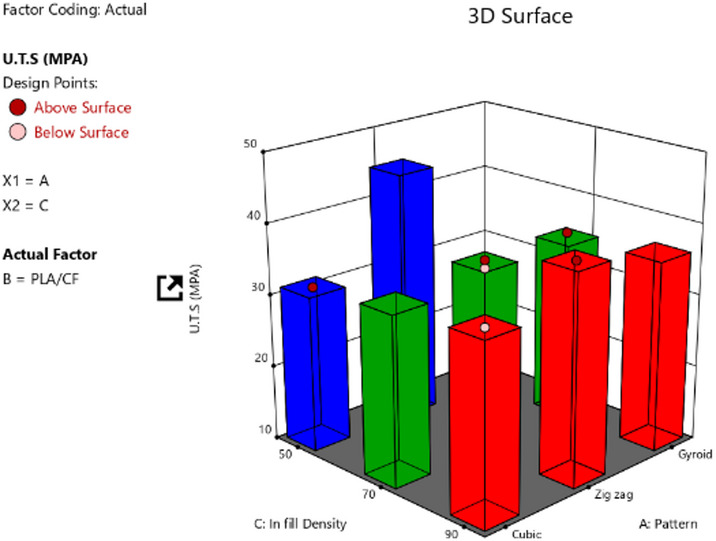
Predicted versus actual values for ultimate tensile strength (heat-treated specimen).

**Fig. 17:**
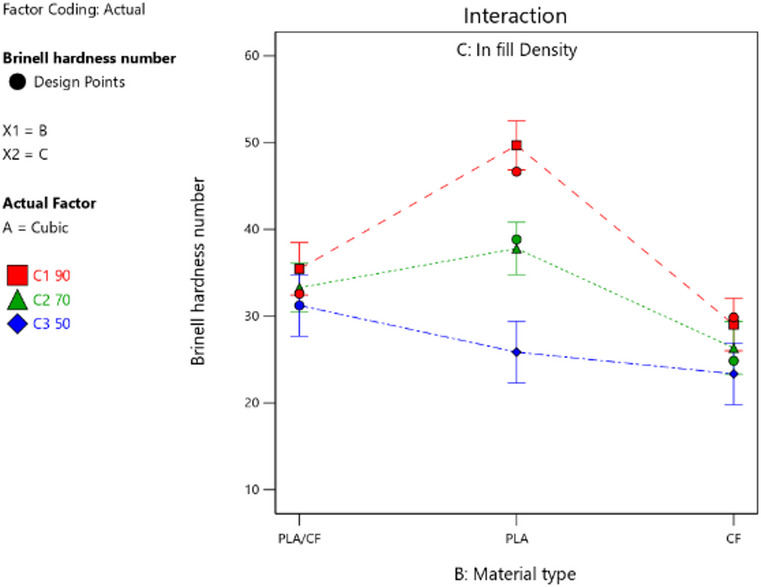
Normal probability plot of residuals for Brinell hardness (heat-treated specimens).

**Fig. 18:**
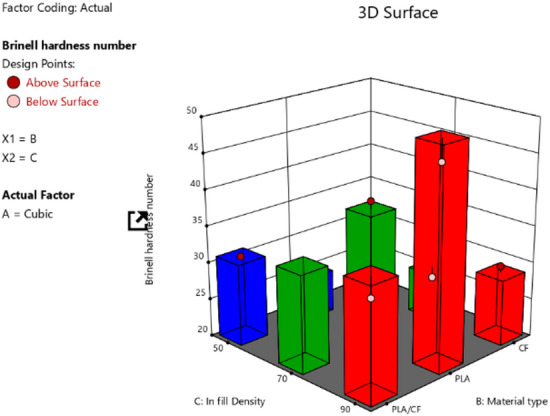
Predicted versus actual values for Brinell hardness (heat-treated specimens).

Future work should explore fatigue performance, thermal cycling effects, and scalability to different geometric sizes and industrial applications (Figs. 14, 15, 16, 17, 18).

## Conclusions

The results of this study demonstrate that the mechanical behavior of FDM‑printed PLA and CF‑PLA components is predominantly governed by material type and infill density, while infill pattern and parameter interactions play secondary yet critical roles. The extensive property ranges observed across the investigated configurations highlight the design flexibility enabled by the strategic selection of processing parameters.

Consistent with the statistical analysis, the following conclusions are drawn:

1. Tensile Strength and Hardness: These properties exhibit a wide variation (up to 147% for UTS), being strongly influenced by material type, infill density, and their interactions. Carbon‑fiber reinforcement significantly enhances the load‑bearing capacity, particularly when paired with high infill density.
2. Elastic Modulus: The elastic modulus demonstrated limited sensitivity to the studied printing parameters and thermal annealing. Statistical analysis (ANOVA) confirmed that the observed variations in stiffness were primarily governed by the bulk material properties and global porosity levels, rather than the internal infill geometry or post‑processing conditions.
3. Thermal Annealing Effects: Post‑printing heat treatment induces variable responses depending on the initial printing state. While modest increases in tensile strength (up to 13%) were observed in specific CF‑PLA configurations, annealing proved most effective for specimens with suboptimal initial conditions. The process facilitates molecular diffusion across filament boundaries, transitioning the failure mode from brittle inter‑bead separation to a more cohesive mechanism.
4. Failure Mechanism: Microscopy and fracture analysis reveal that annealing promotes ‘inter‑bead welding,’ reducing micro‑voids and enhancing interfacial bonding. In CF‑PLA, the fibers act as bridging elements that resist crack propagation, correlating with the improved mechanical integrity observed after thermal treatment.

While this study provides a comprehensive optimization of the annealing process for PLA and CF‑PLA, certain limitations warrant further investigation. The current findings are specific to the material systems and the FDM parameters explored herein; however, the methodology could be extended to other semi‑crystalline polymers like PETG or Nylon to evaluate their specific ‘annealing windows.’ Future research should focus on the long‑term fatigue behavior and creep resistance of these annealed composites under dynamic loading conditions. Additionally, investigating the impact of varying fiber weight fractions and lengths could provide deeper insights into the synergistic effects of reinforcement and thermal treatment, further pushing the boundaries of high‑performance bio‑based additive manufacturing.

## Data Availability

No datasets were generated or analysed during the current study.
